# Do Caregivers Decrease Depression in People With Disabilities? Cross-Sectional Analysis of a Chilean Population-Based Survey

**DOI:** 10.1155/tswj/9018645

**Published:** 2025-09-30

**Authors:** Claudia Andrea Ramos-Carrillo, Maria Fernanda Torres-Marocho, J. Jhonnel Alarco

**Affiliations:** Disability Epidemiology Research Group (EpiDIS), School of Medicine, Universidad Científica del Sur, Lima, Peru

**Keywords:** caregivers, depression, disabled persons, surveys and questionnaires, vulnerable populations

## Abstract

**Objective:**

Although caregiver assistance is generally assumed to improve the mental health of individuals with disabilities, this relationship has not been evaluated using population-based data. This study was aimed at assessing the association between caregiver assistance and depression among individuals with disabilities in Chile in 2015.

**Methods:**

A cross-sectional secondary data analysis was conducted using data from the 2015 Second National Study on Disability (ENDISC II) in Chile. The primary outcome was depression diagnosed by a physician or health professional. The main exposure variable was caregiver assistance. Additional confounding variables were included in the analysis.

**Results:**

The analysis included data from 2610 individuals with disabilities. In the overall sample, caregiver assistance was not associated with depression. However, stratified analysis by disability severity showed that among individuals with severe disabilities, caregiver assistance was associated with a 27% reduction in the prevalence of depression (PR = 0.73; 95% CI: 0.59–0.89), after adjusting for confounding variables.

**Conclusions:**

Among individuals with severe disabilities in Chile, caregiver assistance appears to reduce the likelihood of depression. Healthcare policies should promote and strengthen both formal and informal caregiving for this vulnerable population.

## 1. Introduction

Disability affects approximately 16% of the global population, and its prevalence continues to rise due to population aging and the increasing burden of chronic diseases [1]. In Latin America, prevalence ranges from 6.4% in Colombia to 14.5% in Brazil [2]. In Chile, according to the National Disability Survey, prevalence reached 16.7%, corresponding to 2.8 million individuals [3].

Caregivers—both formal and informal—play a critical role in supporting individuals with disabilities by assisting with activities of daily living and providing emotional and social support [4–7]. Although numerous studies have examined caregiver burden and its impact on the quality of life of those who assume this role [5, 8], there is limited evidence regarding the impact of caregiver support on the mental health of care recipients.

Individuals with disabilities exhibit a higher prevalence of depression compared with the general population [9–11]. However, it remains unclear whether caregiver assistance reduces this risk, particularly in the Latin American context. Therefore, the following research question was posed: Do caregivers reduce depression among individuals with disabilities in Chile? The hypothesis was that caregiver assistance may reduce the prevalence of depression in this population. Additionally, this potential association was evaluated across different levels of disability.

## 2. Methods

### 2.1. Design and Population

We conducted a cross-sectional study using secondary data from the 2015 National Disability Survey II (ENDISC II), carried out by the National Institute of Statistics (INE) of Chile. The primary objective of ENDISC II was to determine the prevalence and main characteristics of disability, as well as to identify access gaps affecting this population group [12]. The preparation of this manuscript adhered to the STROBE guidelines for observational studies [13].

### 2.2. Source of Data

The ENDISC II is a population-based survey specifically designed to assess the disability situation in Chile. It was administered to children aged 2–17 years and adults aged 18 years or older residing in urban and rural areas across 135 municipalities in the 15 regions of the country. The final sampling unit was the household. The sample size for ENDISC II was 11,981 dwellings (equivalent to 12,265 households), calculated based on a previously reported disability prevalence of 12.9%. The survey included 12,265 adults aged 18 years or older and 5515 children and adolescents aged 2–17 years. A probabilistic, two-phase, stratified sampling design was employed, with stratification by municipality and geographic area, ensuring national, regional, and domain-level (urban and rural) representativeness. Data collection was conducted through face-to-face interviews using paper questionnaires, and fieldwork took place between June 30 and September 4, 2015 [12].

### 2.3. Measurement of Disability

ENDISC II measures disability using the Model Disability Survey (MDS), as recommended by the World Health Organization and the World Bank [14]. The MDS represents the current international standard for disability data collection and has been implemented in multiple countries, including Chile [15]. A short version of the MDS has demonstrated robust psychometric properties for assessing levels of disability [16]. This methodology is grounded in the concepts of capacity and functioning defined by the International Classification of Functioning, Disability and Health (ICF) and is supported by evidence from 179 surveys conducted globally [12].

### 2.4. Selection of Participants

For this study, only data from adults aged 18 years or older who presented with some form of disability (*n* = 2618) were included. Records with missing or inconsistent data were excluded.

### 2.5. Variables

Depression was the primary outcome variable and was assessed using two questions: “Has a doctor (or other health professional) ever told you that you have depression?”—indicating a formal diagnosis by a healthcare provider—and “In the last 12 months, have you received any medication for depression?”—indicating recent treatment. Individuals who responded affirmatively to both questions were classified as having depression. This variable was dichotomized with two response categories: yes or no. A similar approach to measuring depression has been used in a previous study [17].

Caregiver assistance was the exposure variable and was assessed with the following question: “Because of your health, do you have someone to help you at home or outside your home—including family and friends—with the following activities: walking or climbing steps, grooming or dressing, using the toilet, getting in and out of bed, doing household chores, caring for or supporting others, going outside, shopping, or going to the doctor?” An individual was considered to receive caregiver assistance if they responded affirmatively to at least one of these activities. For the descriptive and bivariate analyses, responses were classified into three categories (no assistance, informal assistance, and formal assistance), whereas for the multivariate analysis, responses were dichotomized into two categories (no vs. yes).

### 2.6. Covariates

Sociodemographic variables included sex (male or female), age (< 65 or ≥ 65 years), educational level (no formal education, elementary, middle, or high school), marital status (married or cohabiting; separated, divorced, or widowed; and single), and area of residence (urban or rural). Potential confounding variables were also included: indigenous identification [18] assessed with the question “in Chile, the law recognizes nine Indigenous peoples. Do you belong to or are you a descendant of any of them?” (response categories: yes or no); engagement in recreational activities [19] assessed with the question “during the last six months, did you participate in or attend any of the following activities or places?” (yes or no); presence of chronic illness [20], including diabetes, hypertension, arthritis/arthrosis, heart disease, respiratory disease, migraine, and AIDS/HIV, defined as diagnosed by a health professional and treated within the past 12 months (yes or no); and use of rehabilitation services [21] assessed with the question “in the past 12 months, did you receive any rehabilitation services?” (yes or no).

### 2.7. Statistical Processing and Analysis

The database and codebook were obtained from the Chilean National Disability Service (SENADIS) website (https://www.senadis.gob.cl/pag/356/1625/base_de_datos) and imported into Stata 17 software (StataCorp, College Station, Texas, United States) for statistical analysis.

Categorical variables were reported as frequencies, weighted percentages, and 95% confidence intervals (95% CIs). The chi-square test, adjusted for the survey design, was used to assess associations between covariates and caregiver assistance. For the multivariate analysis, a hierarchical approach using three Poisson regression models was employed to estimate the association between caregiver assistance and depression or anxiety. Model 1 was adjusted for sociodemographic variables (sex, age, educational level, marital status, and area of residence); Model 2 included variables from Model 1 plus indigenous identification and engagement in recreational activities; and Model 3 included variables from Model 2 plus chronic illness and use of rehabilitation services. These models were applied across three analytical subgroups: all individuals with disabilities, individuals with mild to moderate disabilities, and individuals with severe disabilities. Forest plots were generated to illustrate the results. Prevalence ratios (PRs) and their corresponding 95% CI were calculated, with a *p* value < 0.05 considered statistically significant. The complex sampling design of ENDISC II was incorporated in all analyses. The variable “VARUNIT_N” identified clusters, “VARSTRAT_N” identified strata, and the expansion factor “Factor_Persona” was used to define the sampling weights. Stata's “subpop” command was applied to analyze subpopulations, ensuring that all proportions were weighted and extrapolated to represent the Chilean population.

### 2.8. Ethical Approval

The study protocol was reviewed and approved by the Institutional Research Ethics Committee of Universidad Científica del Sur (300-2021-PRE15). The ENDISC II dataset is anonymized and publicly available.

## 3. Results

Data from 2618 individuals with disabilities were included. Eight participants (0.3%) were excluded due to missing data (educational level and indigenous identification), resulting in a final analytical sample of 2610 individuals. Among them, 1522 (58.4%) reported mild to moderate disability, while 1088 (41.6%) reported severe disability.

The majority of participants were women (64.3%), under 65 years of age (61.7%), had attained a basic level of education (39.3%), were married or cohabiting (54.0%), and resided in urban areas (86.5%). Additionally, 7.4% self-identified as Indigenous, 74.7% engaged in recreational activities, 74.2% had a chronic illness, and 19.1% had received rehabilitation services within the past 12 months. Overall, 41.2% (95% CI: 39.4–44.0; *n* = 1050) of individuals with disabilities reported receiving assistance from a caregiver. Among these, 38.6% (*n* = 969) received care from informal caregivers and 2.6% (*n* = 86) from formal caregivers. Furthermore, 63.0% (*n* = 724) of caregivers were women, with a mean age of 65.3 years (SE: 0.8). In addition, 27.5% (95% CI: 25.1–29.9) of participants reported having been diagnosed with depression by a physician or other health professional in the past 12 months (Table 1). In the bivariate analysis, no statistically significant association was observed between caregiver assistance and depression (*p* = 0.832) (Table 2).

**Figure 1:**
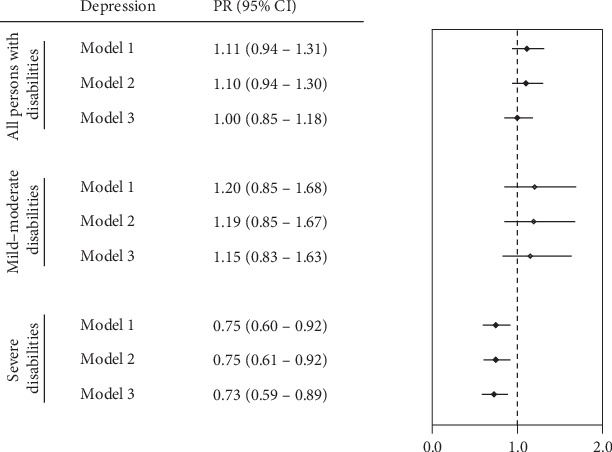
Association between caregiver assistance and depression across levels of disability severity, based on adjusted Poisson regression models. Estimates are presented for the total population with disabilities, individuals with mild to moderate disabilities, and individuals with severe disabilities, according to Models 1, 2, and 3. Model 1: adjusted for sociodemographic variables (sex, age, educational level, marital status, and area of residence). Model 2: model 1 + indigenous identification and recreational activities. Model 3: Model 2 + chronic illness and rehabilitation.

In the multivariate analysis, no significant association was observed between caregiver assistance and depression among the overall population with disabilities or among those with mild to moderate disabilities. However, a significant association was identified among individuals with severe disabilities across all three models. Model 1, adjusted for sociodemographic variables, and Model 2, additionally adjusted for indigenous identification and engagement in recreational activities, yielded similar estimates (PR = 0.75; 95% CI: 0.60–0.92). In Model 3, which adjusted for all covariates (sex, age, educational level, marital status, area of residence, indigenous identification, recreational activities, chronic illness, and rehabilitation), individuals with severe disabilities who received caregiver assistance were 27% less likely to report depression compared with those who did not receive such assistance (PR = 0.73; 95% CI: 0.59–0.89) (Figure 1 and Tables S1–S3).

## 4. Discussion

More than one-third of individuals with disabilities reported receiving assistance from a caregiver. Additionally, one in four reported symptoms of depression. No significant association was observed between caregiver assistance and depression in the overall population of individuals with disabilities. However, when stratified by level of disability, individuals with severe disabilities who received caregiver assistance were less likely to report depression.

A total of 41.2% of individuals with disabilities reported receiving assistance from a caregiver. A comparable figure was observed in a population-based study conducted in Peru, which included 37,117 individuals with disabilities; in that study, 40.5% were reported to be dependent on a caregiver, and 94.3% of them were women [22]. However, these percentages may vary by age group [23] and type of disability [24]. Estimating the proportion of individuals with disabilities who require caregiver support is essential for informing public health policy. Nevertheless, accurate measurement remains challenging due to the absence of standardized definitions for both “caregiver” and “disability” [25].

Depression was identified in 27.5% of individuals with disabilities, a figure consistent with findings from similar studies. A population-based study of 2776 individuals with disabilities in South Korea reported a prevalence of depressive symptoms of 17.7%, although this rate and the associated factors varied by type of disability [26]. Similarly, a study conducted in Shanghai, China, involving 1815 individuals with disabilities, found a prevalence of 23.4% [27]. Some population-based studies have reported higher rates; for instance, in Peru, the prevalence of depression was 50.1% among individuals with disabilities, compared to 30.9% in those without disabilities [28]. This variability may be attributed to differences in the instruments used to assess depression and anxiety, as well as contextual factors such as socioeconomic status and cultural characteristics of the study populations.

No association was observed between caregiver assistance and depression when the analysis included all individuals with disabilities. This highlights the complexity of measuring disability in population-based studies. The dichotomous classification of individuals as either “with disabilities” or “without disabilities” has notable limitations, as it does not capture the full spectrum of disability severity [29]. A more informative approach involves assessing disability by severity level; for example, access to influenza and pneumonia immunizations has been shown to vary with the level of disability, with individuals with severe disabilities experiencing reduced access [30]. Unfortunately, many population-based surveys do not include questions regarding disability severity, which significantly limits the ability to identify critical differences when analyzing the diverse circumstances affecting individuals with disabilities.

When stratified by level of disability, individuals with severe disabilities who received caregiver assistance were less likely to experience depression than those who did not receive such support. Several studies have documented the benefits of caregiver assistance. For example, a study involving 24,036 Canadians with disabilities reported that individuals who received help with household tasks or assistance from family members, friends, or specialized agencies were more likely to report happiness than those who received no help [31]. Among 610 HIV-infected adults in Rwanda, the presence of a companion was associated with a 44.3% reduction in depression prevalence, as well as improvements in quality of life and social support during the first year of treatment [32]. A meta-analysis of 148 studies found that the presence of social relationships increased the likelihood of survival by 50% [33]. Although these findings support our results, the overall evidence remains limited and outcome-dependent. For instance, caregiver assistance was not associated with improved weight control among individuals with intellectual disabilities [34] or with reduced mortality risk in older adults [35].

The potential protective effect of caregiver assistance against depression can be explained through several mechanisms. Caregivers provide continuous companionship, which reduces loneliness and fosters a sense of security—both factors strongly associated with better mental health [33]. They also contribute to emotional regulation through empathy, encouragement, and reassurance, which are particularly valuable for individuals with severe functional limitations [32, 36]. In addition, the presence of a caregiver promotes social connectedness and facilitates adherence to treatment, participation in community activities, and interaction with healthcare services, thereby enhancing self-efficacy and perceived autonomy [31, 37, 38]. These mechanisms are consistent with the literature on social support, which consistently demonstrates that supportive relationships reduce the risk of depression and improve well-being in vulnerable populations [9, 33]. Therefore, our findings support the hypothesis that caregiver assistance not only provides practical help with activities of daily living but also constitutes an essential form of emotional and social support that protects against depression among individuals with severe disabilities.

It is essential to implement policies that promote and fund home-based care programs focused on individuals with severe disabilities, including support for informal caregivers through training and psychosocial counseling. Additionally, caregiver involvement and the assessment of the “care environment” should be integrated into medical care, identifying the presence, availability, and quality of the support provided. This approach is aimed at guiding targeted interventions to improve patient health or to facilitate timely referrals to community services when support is inadequate.

It is important to note that the cross-sectional design precludes establishing causal relationships. Although our results suggest a protective association between caregiver assistance and depression, alternative explanations cannot be ruled out. For instance, individuals with fewer depressive symptoms may have a greater capacity to sustain strong family and social ties, thereby facilitating access to caregiver support. Conversely, families may be more inclined to provide assistance to members with better mental health, creating the possibility of reverse causality. Prior literature has highlighted that the relationship between social support and depression can be bidirectional, as depression may erode social networks, whereas the presence of social connections protects against depressive symptoms [39–41]. In addition, unmeasured factors such as household economic resources, family environment quality, or access to healthcare services may confound the observed association. Therefore, longitudinal studies are needed to clarify the directionality of these relationships and to better understand the causal mechanisms linking caregiver assistance with mental health.

### 4.1. Limitations and Strengths

As this study is based on secondary data analysis, several limitations must be acknowledged. Depression was defined as the combination of self-reported lifetime medical diagnosis and the use of antidepressant medication in the past 12 months. Although this approach has been widely applied in epidemiological surveys [42–44], it presents several challenges. First, underdiagnosis of depression is common among individuals with disabilities, which may lead to an underestimation of its true prevalence. Second, recall bias and social desirability bias may have influenced self-reported responses. Third, the question “has a doctor (or other health professional) ever told you that you have depression?” does not ensure that the respondent was experiencing depression at the time of the interview, which could result in an overestimation of prevalence [45]. To mitigate this limitation, the use of antidepressant medication in the previous year was included as a proxy for current depressive status, thereby increasing the specificity of our definition. Nonetheless, future studies should incorporate validated instruments such as the PHQ-8 or PHQ-9, which have demonstrated greater accuracy in identifying current depressive symptoms [46].

It is important to consider the potential for selection bias. Individuals with severe disabilities who receive caregiver assistance may systematically differ from those who do not, for example, by having stronger family networks, better socioeconomic resources, or greater access to social and healthcare services. Such differences could bias the observed association between caregiver assistance and depression. In addition, unmeasured confounders—such as household income, quality of family relationships, or history of mental health conditions—were not captured in the ENDISC II database and may partly account for the associations identified. Although our models were adjusted for several relevant covariates, the possibility of residual confounding cannot be excluded. Future studies should incorporate broader measures of social support, family environment, and economic circumstances to more accurately assess the effect of caregiver assistance on mental health outcomes among individuals with disabilities.

Another important limitation relates to the timeliness of the data analyzed. The ENDISC II survey was conducted in 2015, which may not fully reflect the current disability and healthcare landscape in Chile. Although a more recent survey (ENDISC III, 2022) has been announced, its microdata are not yet publicly available through the official SENADIS website. Despite this limitation, to the best of our knowledge, no other population-based studies have evaluated the relationship between caregiver assistance and depression among people with disabilities in Chile. Therefore, these findings represent an initial contribution to a scarcely studied topic in Latin America and may serve as a foundational reference for future research using updated data. Due to the cross-sectional design of the study, causal relationships among the primary variables cannot be established. Nevertheless, the population-based nature of the survey enhances the generalizability of the findings to the broader population of individuals with disabilities in Chile.

### 4.2. Conclusions

This study demonstrates that while caregiver assistance is not associated with a lower prevalence of depression among all individuals with disabilities, it does exert a significant protective effect among those with severe disabilities, reducing the probability of depression by 27% after adjusting for confounding factors. This finding underscores the importance of continuous support for individuals with greater functional limitations and suggests that daily companionship, emotional support, and assistance with basic activities provided by caregivers may play a key role in the mental health of this population. Public health policies should promote home-based care for individuals with severe disabilities, and clinical practice should encourage caregiver involvement to guide targeted interventions for this vulnerable population group.

## Figures and Tables

**Table 1:** Characteristics of people with disabilities in Chile (*n* = 2610).

**Variables**	**n**	**% ** ^**†**^	**95% CI**
Sex			
Male	801	35.7	33.0–38.4
Female	1809	64.3	61.6–67.0
Age			
< 65 years	1524	61.7	58.8–64.6
≥ 65 years	1086	38.3	35.4–41.2
Educational level			
No education	191	7.5	6.2–9.0
Elementary	1077	39.3	36.6–42.2
Middle	986	38.2	35.7–40.7
High school	356	15.0	13.0–17.3
Marital status			
Married or cohabiting	1181	54.0	51.4–56.5
Separated or divorced or widowed	841	24.8	22.6–27.2
Single	588	21.2	19.1–23.5
Area of residence			
Urban	2176	86.5	84.1–88.6
Rural	434	13.5	11.4–15.6
Indigenous identification			
No	2388	92.6	91.1–93.9
Yes	222	7.4	6.1–8.9
Chronic illness			
No	633	25.8	23.4–28.3
Yes	1977	74.2	71.7–76.6
Rehabilitation			
No	2107	80.9	78.7–82.9
Yes	503	19.1	17.1–21.3
Recreational activities			
No	639	25.3	22.9–27.8
Yes	1971	74.7	72.2–77.1
Caregiver assistance			
No	1560	58.8	55.9–61.6
Informal	969	38,6	35.7–41.5
Formal	81	2,6	1.8–3.8
Depression			
No	1892	72.5	70.0–74.9
Yes	718	27.5	25.1–29.9

^†^Percentage weighted according to ENDISC II complex sampling.

**Table 2:** Characteristics of people with disabilities in Chile according to diagnosis of depression (*n* = 2610).

**Variables**	**Depression**		**p**
	**No**	**Yes**	
Sex			< 0.001
Male	654 (82.2)	147 (17.8)	
Female	1238 (67.2)	571 (32.8)	
Age			0.218
< 65 years	1066 (71.3)	458 (28.7)	
≥ 65 years	826 (74.5)	260 (25.5)	
Educational level			0.708
No education	155 (77.2)	36 (22.8)	
Elementary	781 (71.5)	296 (28.5)	
Middle	704 (72.9)	282 (27.1)	
High school	252 (71.7)	104 (28.3)	
Marital status			0.365
Married or cohabiting	864 (73.2)	317 (26.8)	
Separated or divorced or widowed	603 (69.5)	238 (30.5)	
Single	425 (74.4)	163 (25.6)	
Area of residence			0.154
Urban	1546 (71.7)	630 (28.3)	
Rural	346 (77.7)	88 (22.3)	
Indigenous identification			0.346
No	1728 (72.2)	660 (27.8)	
Yes	164 (76.3)	58 (23.7)	
Chronic illness			< 0.001
No	516 (83.3)	117 (16.7)	
Yes	1376 (68.8)	601 (31.2)	
Rehabilitation			< 0.001
No	1601 (75.9)	506 (24.1)	
Yes	291 (58.0)	212 (42.0)	
Recreational activities			0.952
No	457 (72.7)	182 (27.3)	
Yes	1435 (72.5)	536 (27.5)	
Caregiver assistance			0.778
No	1129 (72.7)	431 (27.3)	
Informal	706 (71.9)	263 (28.1)	
Formal	57 (76.7)	24 (23.3)	

*Note:* The complex sampling of the ENDISC II was considered in all calculations.

## Data Availability

ENDISC II data is available on the website of the National Disability Service (SENADIS) of Chile: https://www.senadis.gob.cl/pag/356/1625/base_de_datos.

## Supporting Information

Supporting Information Additional supporting information can be found online in the Supporting Information section. Table S1 presents the multivariable models calculated for all individuals with disabilities (*n* = 2610). Table S2 presents the multivariable models calculated only for individuals with mild–moderate disability (*n* = 1522). Table S3 presents the multivariable models calculated only for individuals with severe disability (*n* = 1088).
