# Supplementary material for: Do Caregivers Decrease Depression in People With Disabilities? Cross-Sectional Analysis of a Chilean Population-Based Survey
Source: ScientificWorldJournal. 2025 Sep 30;2025:9018645. doi: 10.1155/tswj/9018645 (PMC12503989; doi:10.1155/tswj/9018645)
Supplement: Supporting Information — Additional supporting information can be found online in the Supporting Information section. Table S1 presents the multivariable models calculated for all individuals with disabilities (n = 2610). Table S2 presents the multivariable models calculated only for individuals with mild–moderate disability (n = 1522). Table S3 presents the multivariable models calculated only for individuals with severe disability (n = 1088). [file 9018645.f1.docx]

**Supplementary Material**

**Table S1.** Adjusted models for all disabled people with depression (n=2610).

| **Variables** | **Model 1** | **Model 2** | **Model 3** |
| --- | --- | --- | --- |
|  | **PR (IC 95%)** | **PR (IC 95%)** | **PR (IC 95%)** |
| **Caregiver** |  |  |  |
| No | 1 | 1 | 1 |
| Yes | 1.11 (0.94‒1.31) | 1.10 (0.94‒1.30) | 1.00 (0.85‒1.18) |
| **Sex** |  |  |  |
| Male | 1 | 1 | 1 |
| Female | 1.82 (1.44‒2.31) | 1.83 (1.45‒2.32) | 1.71 (1.36‒2.15) |
| **Age** |  |  |  |
| < 65 years | 1 | 1 | 1 |
| ≥ 65 years | 0.84 (0.68‒1.03) | 0.83 (0.67‒1.02) | 0.76 (0.62‒0.94) |
| **Educational level** |  |  |  |
| No education | 1 | 1 | 1 |
| Elementary | 1.24 (0.82‒1.87) | 1.23 (0.81‒1.86) | 1.11 (0.75‒1.64) |
| Middle | 1.16 (0.76‒1.75) | 1.15 (0.76‒1.74) | 1.06 (0.71‒1.57) |
| High school | 1.26 (0.80‒1.98) | 1.25 (0.80‒1.97) | 1.21 (0.79‒1.85) |
| **Marital status** |  |  |  |
| Married or cohabiting | 1 | 1 | 1 |
| Separated or divorced or widowed | 1.03 (0.83‒1.28) | 1.02 (0.83‒1.26) | 1.02 (0.82‒1.27) |
| Single | 0.91 (0.71‒1.16) | 0.91 (0.71‒1.16) | 1.01 (0.80‒1.26) |
| **Origin** |  |  |  |
| Urban | 1 | 1 | 1 |
| Rural | 0.82 (0.58‒1.16) | 0.83 (0.58‒1.17) | 0.84 (0.60‒1.17) |
| **Indigenous identification** |  |  |  |
| No |  | 1 | 1 |
| Yes |  | 0.84 (0.60‒1.18) | 0.90 (0.65‒1.26) |
| **Recreational activities** |  |  |  |
| No |  | 1 | 1 |
| Yes |  | 0.97 (0.77‒1.22) | 0.97 (0.78‒1.21) |
| **Chronic illness** |  |  |  |
| No |  |  | 1 |
| Yes |  |  | 1.88 (1.45‒2.46) |
| **Rehabilitation** |  |  |  |
| No |  |  | 1 |
| Yes |  |  | 1.70 (1.43‒2.03) |

Model 1: adjusted for sociodemographic variables (i.e., sex, age, educational level, marital status, and origin).

Model 2: model 1 + indigenous identification and recreational activities.

Model 3: model 2 + chronic illness and rehabilitation.

**Table S2.** Adjusted models for only persons with mild–moderate disabilities with depression (n=1522).

| **Variables** | **Model 1** | **Model 2** | **Model 3** |
| --- | --- | --- | --- |
|  | **RP (IC 95%)** | **RP (IC 95%)** | **RP (IC 95%)** |
| **Caregiver** |  |  |  |
| No | 1 | 1 | 1 |
| Yes | 1.20 (0.85‒1.68) | 1.19 (0.85‒1.67) | 1.15 (0.83‒1.63) |
| **Sex** |  |  |  |
| Male | 1 | 1 | 1 |
| Female | 1.90 (1.33‒2.72) | 1.91 (1.35‒2.70) | 1.77 (1.24‒2.50) |
| **Age** |  |  |  |
| < 65 years | 1 | 1 | 1 |
| ≥ 65 years | 0.92 (0.66‒1.29) | 0.92 (0.66‒1.29) | 0.80 (0.57‒1.11) |
| **Educational level** |  |  |  |
| No education | 1 | 1 | 1 |
| Elementary | 3.21 (1.42‒7.23) | 3.20 (1.41‒7.22) | 3.08 (1.40‒6.81) |
| Middle | 3.04 (1.36‒6.82) | 3.06 (1.36‒6.88) | 3.09 (1.41‒6.77) |
| High school | 3.80 (1.62‒8.90) | 3.84 (1.63‒9.07) | 4.13 (1.80‒9.48) |
| **Marital status** |  |  |  |
| Married or cohabiting | 1 | 1 | 1 |
| Separated or divorced or widowed | 1.05 (0.72‒1.51) | 1.04 (0.73‒1.50) | 1.04 (0.71‒1.51) |
| Single | 0.99 (0.71‒1.40) | 1.00 (0.71‒1.40) | 1.12 (0.82‒1.53) |
| **Origin** |  |  |  |
| Urban | 1 | 1 | 1 |
| Rural | 0.85 (0.48‒1.49) | 0.85 (0.48‒1.49) | 0.90 (0.51‒1.56) |
| **Indigenous identification** |  |  |  |
| No |  | 1 | 1 |
| Yes |  | 0.99 (0.60‒1.62) | 1.08 (0.67‒1.73) |
| **Recreational activities** |  |  |  |
| No |  | 1 | 1 |
| Yes |  | 0.94 (0.63‒1.38) | 0.94 (0.64‒1.37) |
| **Chronic illness** |  |  |  |
| No |  |  | 1 |
| Yes |  |  | 2.05 (1.45‒2.91) |
| **Rehabilitation** |  |  |  |
| No |  |  | 1 |
| Yes |  |  | 1.53 (1.16‒2.03) |

Model 1: adjusted for sociodemographic variables (i.e., sex, age, educational level, marital status, and origin).

Model 2: model 1 + indigenous identification and recreational activities.

Model 3: model 2 + chronic illness and rehabilitation.

**Table S3.** Adjusted models for only persons with severe disabilities with depression (n=1088).

| **Variable** | **Model 1** | **Model 2** | **Model 3** |
| --- | --- | --- | --- |
|  | **PR (IC 95%)** | **PR (IC 95%)** | **PR (IC 95%)** |
| **Caregiver** |  |  |  |
| No | 1 | 1 | 1 |
| Yes | 0.75 (0.60‒0.92) | 0.75 (0.61‒0.92) | 0.73 (0.59‒0.89) |
| **Sex** |  |  |  |
| Male | 1 | 1 | 1 |
| Female | 1.78 (1.34‒2.35) | 1.77 (1.34‒2.35) | 1.65 (1.26‒2.17) |
| **Age** |  |  |  |
| < 65 years | 1 | 1 | 1 |
| ≥ 65 years | 0.72 (0.56‒0.92) | 0.72 (0.56‒0.91) | 0.71 (0.56‒0.90) |
| **Educational level** |  |  |  |
| No education | 1 | 1 | 1 |
| Elementary | 0.98 (0.63‒1.54) | 0.97 (0.62‒1.51) | 0.88 (0.57‒1.36) |
| Middle | 1.01 (0.63‒1.62) | 0.99 (0.61‒1.58) | 0.87 (0.55‒1.37) |
| High school | 0.86 (0.49‒1.48) | 0.83 (0.48‒1.42) | 0.76 (0.45‒1.28) |
| **Marital status** |  |  |  |
| Married or cohabiting | 1 | 1 | 1 |
| Separated or divorced or widowed | 0.95 (0.75‒1.22) | 0.95 (0.75‒1.21) | 0.99 (0.77‒1.26) |
| Single | 0.77 (0.56‒1.07) | 0.78 (0.56‒1.07) | 0.87 (0.65‒1.16) |
| **Origin** |  |  |  |
| Urban | 1 | 1 | 1 |
| Rural | 0.84 (0.60‒1.19) | 0.86 (0.62‒1.21) | 0.80 (0.57‒1.12) |
| **Indigenous identification** |  |  |  |
| No |  | 1 | 1 |
| Yes |  | 0.71 (0.46‒1.09) | 0.74 (0.50‒1.09) |
| **Recreational activities** |  |  |  |
| No |  | 1 | 1 |
| Yes |  | 1.09 (0.84‒1.41) | 1.06 (0.83‒1.37) |
| **Chronic illness** |  |  |  |
| No |  |  | 1 |
| Yes |  |  | 1.53 (1.03‒2.25) |
| **Rehabilitation** |  |  |  |
| No |  |  | 1 |
| Yes |  |  | 1.70 (1.38‒2.09) |

Model 1: adjusted for sociodemographic variables (i.e., sex, age, educational level, marital status, and origin).

Model 2: model 1 + indigenous identification and recreational activities.

Model 3: model 2 + chronic illness and rehabilitation.
